# Editorial: Inequalities

**DOI:** 10.3389/fsoc.2022.937162

**Published:** 2022-06-28

**Authors:** Hannah Bradby, Guillermina Jasso, William Outhwaite, Valeria Pulignano, Kath Woodward

**Affiliations:** ^1^Department of Sociology, Uppsala University, Uppsala, Sweden; ^2^Department of Sociology, New York University, New York, NY, United States; ^3^Sociology, Newcastle University, Newcastle upon Tyne, United Kingdom; ^4^Centre for Sociological Research, KU Leuven, Leuven, Belgium; ^5^Department of Sociology, The Open University, Milton Keynes, United Kingdom

**Keywords:** overall inequality and subgroup inequality, inequality in quantitative characteristics (like schooling/income/wealth), inequality across qualitative characteristics (like gender/race/ethnicity/nativity/citizenship), effects of qualitative characteristics on own behaviors and others' assessments, inequality and poverty/justice/status/power, intergenerational and intersectionality mechanisms, national and subnational contexts and cultures, inequality mechanisms in politics and social movements

Inequality is a foundational topic for sociology, and a core topic, in one way or another, for all the social sciences. Inequality research, whose origins can be traced to Confucius (551-479 BCE) and Plato (428/427-348/347 BCE), spans theoretical and empirical analyses (1) of inequality itself, such as its properties and measures across the vast domains of the natural and social goods and bads, from schooling, work, and health to burdens and punishments to income and wealth; (2) of the mechanisms that establish, entrench, and transmit it across generations and locations; (3) of the types of inequality, such as inequality between persons and inequality between subgroups, and their link; and (4) of the broader connections among inequality, poverty, and justice, as well as happiness and social cohesion. This Research Topic includes contributions toward understanding the sociological landscape of inequality, along with investigations of the mechanisms that bring about inequalities at the interpersonal level, and more speculative consideration of the macro-processes at policy and population level which allow injurious inequalities to flourish and offer explanations of their endurance.

While inequality can occur in all contexts of category and differentiation, such as race, ethnicity, nativity, religion, sex, gender and sexuality, generation, embodiment, disability, and class, most of the original research in this Research Topic focuses on sex and gender. Below is an indexing of all the contributions, by article type. While disability, immigration, LGBTQI, and racialized identities are mentioned, it is the investigation of gendered roles, expectations, and stereotypes that dominate this snapshot of inequality research. While gender is the most investigated aspect of inequality in this Research Topic of papers, it is also difficult to draw conclusions from across the papers because the range of theoretical and methodological approaches to gender is wide. Gendered aspects of social life, such as occupational attributes and bias in student evaluations are assessed in this Research Topic, alongside an assessment of the performance of sexism scales that were validated decades ago. One of the most original approaches to gender is the investigation of womenhood in Igbo land that teases out how gendered oppression of women persists across time and through generations.

While this Research Topic of papers is not tightly focused, it does illustrate the great potential for emprirical, methodological and theoretical research around the vast inequality landscape, tracing ever more closely how inequality operates and how it increases and decreases. Particularly welcome will be approaches to understanding inequality that pay heed to more than one dimension of inequality, as the non-additive nature of how gender, class, dis/ability, sexuality and racialization play out intersectionally.

## Original Research

Hustad et al. analyze administrative and self-reported data to consider whether occupational attributes are associated with a sex-distribution in Sweden. The proportion of women was shown to be on average higher in occupations classified as “verbally demanding” and “people oriented” and lower in those classified as “numerically demanding” and “things oriented”. During the ten-year period of study (2002-2011) and including all Swedish occupations, the occupational gender segregation declined.

Garcia-Sanchez et al. investigate the psychometric properties of three sexism scales in a sample of 700 undergraduates, to show that men showed greater support for sexist attitudes than women. The authors seek to understand how gender stereotypes and gender role attitudes relate to each other by comparing scores between scales and classifying their sample in terms of stereotype and of attitude. Since gender concepts constantly evolve, sexism scales that are 30 years out of date do not reflect the changing position of women in society and the development of sexism, so need updating.

Villanueva-Moya and Exposito explore how gender roles can contribute to understanding behaviors that are often considered to be gendered, in this case risk-taking. The authors focus on self-reporting from a sample of 417 Spanish adults around their femininity, fear of negative evaluation and social risk taking, to differentiate how gender roles may influence sex-differences in behavior.

Sauer considers what is referred to as the *justice evaluation* of earnings, that is whether or not people evaluated a gender pay gap as legitimate in Germany. A student sample had no gender bias in their evaluation of appropriate pay for men and women, whereas a population survey showed that men and women rated men as more deserving of higher salary. Respondents in settings characterized by high gender pay gaps produced a larger bias favoring men.

Wang and Sakamoto consider intergenerational transmission of education as an indicator of inclusiveness and inequality among Hispanic Americans. Greater educational attainment, when linked to better labor market outcomes is seen as a crucial motor of immigrant integration. Results from this study indicate how the intergenerational transmission of education varies between Hispanic and non-Hispanic White men and women and takes into account contextual demographic characteristics, such as the percentage of college educated people in the county.

Liu et al. investigate pro-social behavior among people with irreversible physical disabilities and people without disabilities, positing people with disabilities as being at a stable and unchanging social disadvantage. A group of 102 men and women from Zhejiang province, China, of which half had disabilities, participated in a “dictator game”, used to test generosity and cooperative strategies. Within the game, compared to people without disabilities, people with disabilities showed more generous and cooperative social strategies, confirming previous studies.

Özgümüs et al. examine gender bias in the evaluation of teaching materials. Through constructing various experiments wherein subjects evaluate a named fictious instructor's lecture slides, the study shows that evaluation bias is not due to differences in class room experience and thereby question how student evaluations are currently used in higher education.

Mitra and Schicktanz offer an analysis of how Alzheimer disease patient support organizations construct patients' rights. By comparing how patient organizations from the United States, Germany and the United Kingdom set out patients' rights in website text, the authors argue that the context of the healthcare regime sets up path dependencies for how citizenship can be constructed. These path dependencies help explain the differential modes of performing or contesting citizenships that the authors describe in the website texts in the three national settings.

## Policy and Practice Review

Donnelly-Drummond considers how exemptions for equality legislation in the UK constitute a form of violence against LGBTQ people. The discussion centers on two cases of religious exemption from sexual orientation discrimination, so as to reinforce heteronormativity in a way that is argued to perpetuate harm against LGBTQ people, such that the exemptions should not be permitted.

## Hypothesis and Theory

Ekweariri gives an in-depth exploration of the meaning and experience of womanhood in Igbo land (south eastern Nigeria) with particular focus on what is implied by women being the property of men. Ekweariri argues that the oppressive depictions of women are not consciously intended by the agents involved, a proposition that has implications for how gendered subservience persists despite the original context of relationships between wife and husband is long gone.

## Commentary

Condon et al. underline that the COVID-19 pandemic risks exacerbating health inequalities, leading to worsening outcomes for families that face discrimination due to their status as Black, indigenous, people of color.

## Review

Ahlberg et al. examine how racism plays out and is exacerbated by neoliberal forms of welfare state reform in an era of globalized migration. As welfare provision is subject to market logics, hiring migrant labor to reduce costs becomes a norm, while racist populism blames immigrants for restrictions in access to welfare services. The shift of power toward global elites implied by the erosion of collective responsibility for welfare provision is part of the difficulty of making racialized power structures visible to critical analysis and allows entrenched inequality to go unremarked upon.

Siltala's review paper considers the origins of the post-financial-crash-of-2008 shift toward right wing populism. Using political psychology, he considers how economic uncertainty can lead people to abandon class-based identification for other forms of identity politics. Conceptualizing populations as risk-averse right-wing authoritarians and social-dominance oriented risk takers, Siltala discusses why identity politics is more useful to right wing movements than left wing movements.

## Opinion

Scambler asserts the primacy of class over other forms of social stratification and division that drive health inequalities. The reduced relevance of class for subjective identities, does not affect the ongoing material, objective, more quantifiable impact. A personal and powerful call to sociologists to stand their ground in interrogating and calling out the harms that capitalist systems perpetuate against the health of the working class.

## Author Contributions

All authors listed have made a substantial, direct, and intellectual contribution to the work and approved it for publication.

## Conflict of Interest

The authors declare that the research was conducted in the absence of any commercial or financial relationships that could be construed as a potential conflict of interest.

## Publisher's Note

All claims expressed in this article are solely those of the authors and do not necessarily represent those of their affiliated organizations, or those of the publisher, the editors and the reviewers. Any product that may be evaluated in this article, or claim that may be made by its manufacturer, is not guaranteed or endorsed by the publisher.

